# Novel Arterial Reconstruction of the Left Gastric Artery Supplying the Replaced Left Hepatic Artery in Distal Pancreatectomy with Celiac Axis Resection

**DOI:** 10.1245/s10434-026-19098-y

**Published:** 2026-01-20

**Authors:** Kosei Takagi, Atene Ito, Tomokazu Fuji, Kazuya Yasui, Takeyoshi Nishiyama, Yasuo Nagai, Shohei Yokoyama, Toshiyoshi Fujiwara

**Affiliations:** Department of Gastroenterological Surgery, Dentistry, and Pharmaceutical Sciences, Okayama University Graduate School of Medicine, Okayama, Japan

**Keywords:** Distal pancreatectomy with celiac axis resection, Arterial reconstruction, Pancreatic cancer

## Abstract

**Background:**

Distal pancreatectomy with celiac axis resection (DP-CAR) with reconstruction of the left gastric artery (LGA) is a technically challenging procedure. The middle colic artery is commonly used for LGA reconstruction. This study highlights our novel arterial reconstruction of the LGA using the common hepatic artery (CHA) supplying the replaced left hepatic artery (rLHA) during DP-CAR.

**Patient and Methods:**

A 65-year-old man diagnosed with locally advanced unresectable pancreatic body cancer underwent DP-CAR following systemic chemotherapy. As a rLHA arising from the LGA was present, arterial reconstruction was necessary.

**Results:**

After confirming resectability, the CHA and LGA were encircled. Following division of the pancreas and radical lymphadenectomy, the origin of the celiac axis (CA) was divided. Subsequently, the CHA and LGA were transected and anastomosed. An indocyanine green fluorescence system was used to confirm adequate arterial blood supply and satisfactory tissue perfusion. Operative time was 215 min, with an estimated blood loss of 35 mL.

**Conclusions:**

This study demonstrated a novel arterial reconstruction of the LGA supplying the rLHA during DP-CAR. The CHA may be a candidate for the reconstruction of the LGA in DP-CAR.

**Supplementary Information:**

The online version contains supplementary material available at 10.1245/s10434-026-19098-y.

Distal pancreatectomy with celiac axis resection (DP-CAR) is a technically demanding procedure to achieve R0 resection in patients with borderline resectable or locally advanced unresectable pancreatic body cancer. Although DP-CAR can expand the resectability of advanced pancreatic cancer, gastric and hepatic ischemias are associated with significant procedure-specific morbidities, potentially leading to serious complications and mortality. While arterial reconstruction is not commonly performed using this procedure, left gastric artery (LGA) reconstruction should be considered for the purpose of preventing ischemic gastropathy whenever possible, regardless of hepatic artery anatomy. Moreover, in cases with the LGA supplying the replaced left hepatic artery (rLHA), arterial reconstruction should be performed. Herein, we demonstrated a novel arterial reconstruction of the LGA supplying the rLHA using the common hepatic artery (CHA) during DP-CAR (Supporting Video 1).

## Case

**Fig. 1:**
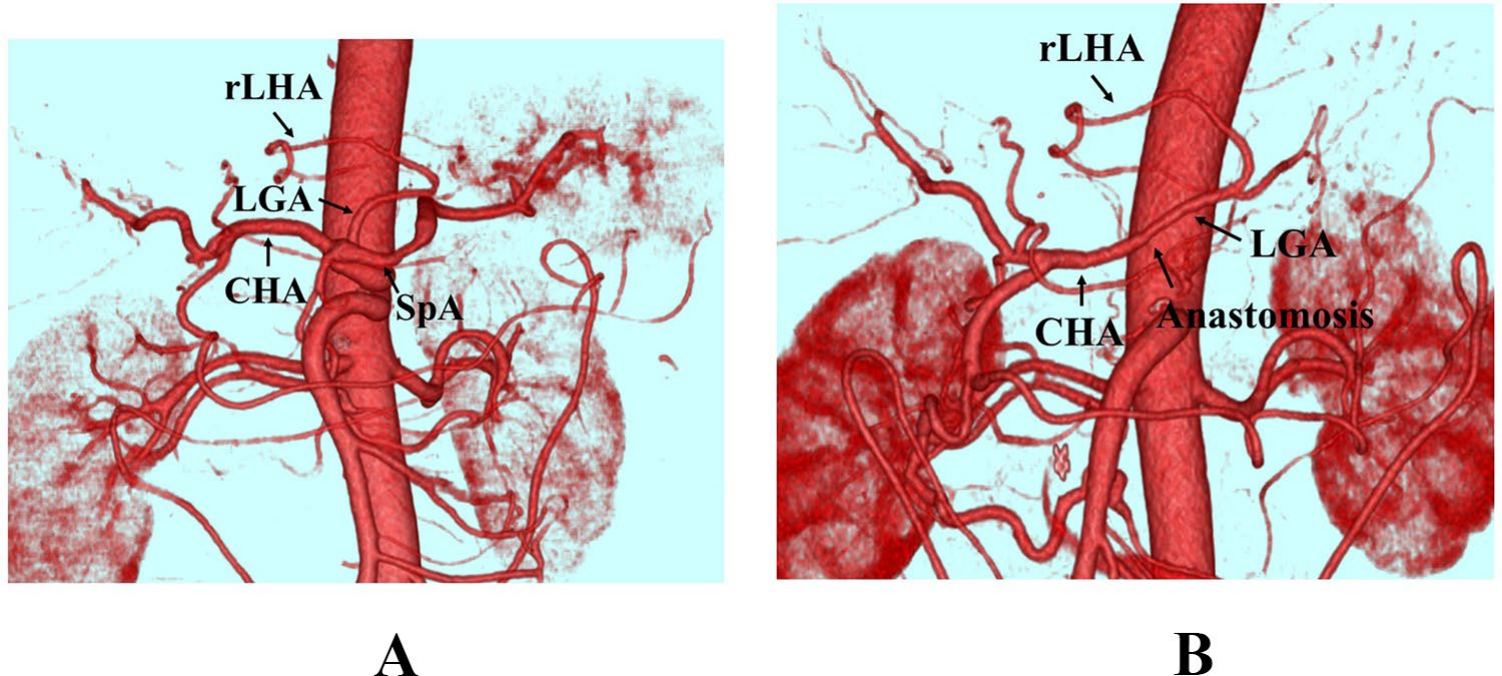
3D computed tomography angiography; **A** preoperative image showing the replaced left hepatic artery (rLHA) arising the left gastric artery (LGA); **B** postoperative image showing patent rLHA and LGA from the common hepatic artery; *CHA* common hepatic artery, *LGA* left gastric artery, *rLHA* replaced left hepatic artery, *SpA* splenic artery

A 65-year-old man diagnosed with locally advanced, unresectable pancreatic body cancer was referred to our institution. The patient underwent DP-CAR as conversion surgery following systemic chemotherapy with gemcitabine and nab-paclitaxel. Preoperative computed tomography revealed a 25-mm tumor invading the bifurcation of the CHA, LGA, and splenic artery from the celiac axis (CA). The three-dimensional (3D) image demonstrated the rLHA arising from the LGA (Fig. 1A). As the LGA root was not preservable, arterial reconstruction was required to ensure hepatic and gastric blood flow.

## Surgical Technique

**Fig. 2:**
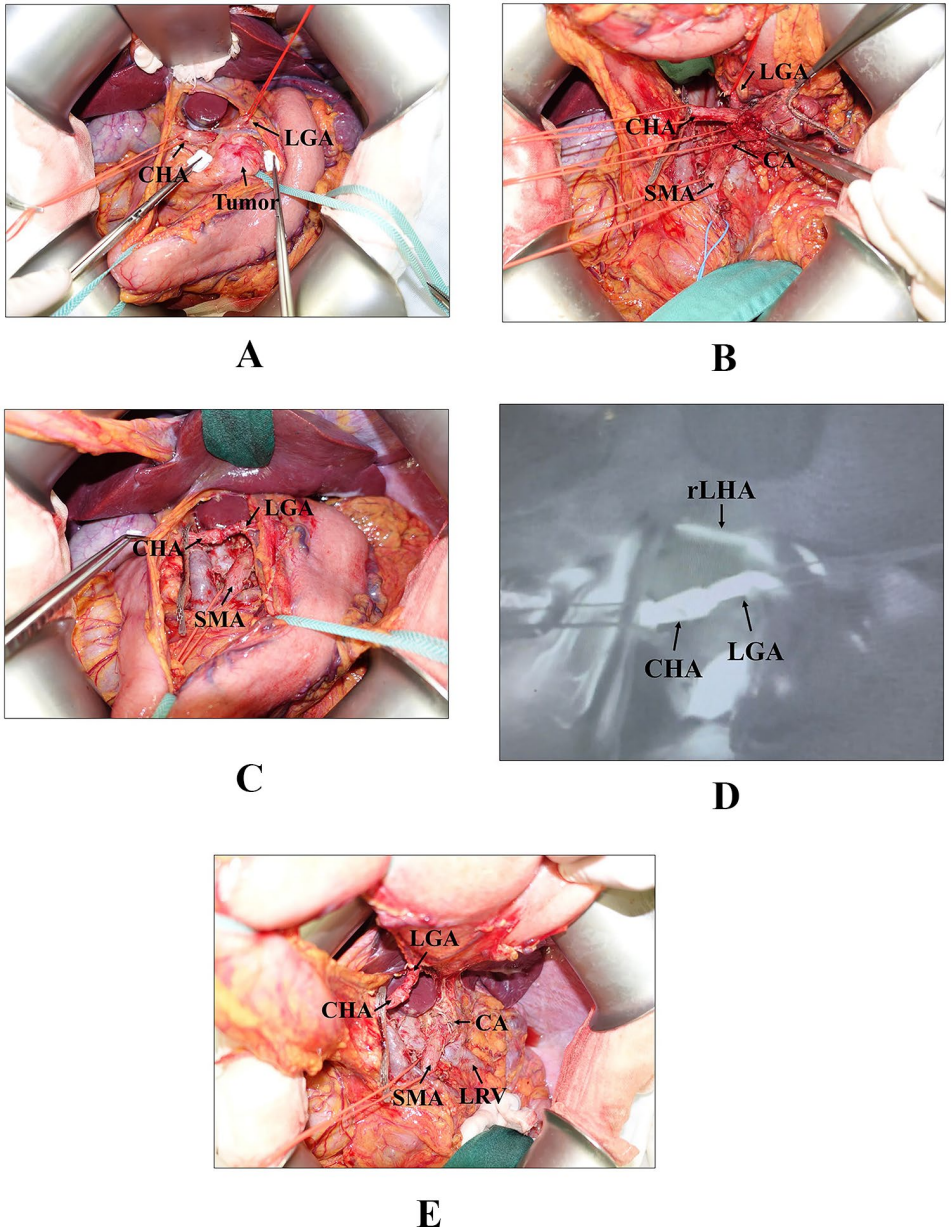
Intraoperative findings; **A** following the dissection of the gastrohepatic ligament, the common hepatic artery (CHA) and left gastric artery (LGA) were encircled, and a tumor invaded the bifurcation of the CHA, LGA, and splenic artery; **B** lymphadenectomy around the major vessels, along with retroperitoneal dissection was performed; **C** following the division of CHA, LGA, and CA, arterial reconstruction between the CHA and LGA was performed with a running end-to-end anastomosis; **D** intraoperative angiography with indocyanine green-fluorescence imaging confirmed good arterial perfusion of reconstructed artery; and **E** overview following a novel arterial reconstruction of the LGA supplying the replaced left hepatic artery in distal pancreatectomy with celiac axis resection; *CA* celiac axis, *LRV* left renal vein, *SMA* superior mesenteric artery

Following confirmation of the absence of peritoneal dissemination or liver metastasis using staging laparoscopy, a laparotomy was performed. The gastrohepatic ligament was dissected to encircle the CHA and LGA (Fig. 2A). The superior border of the pancreas was dissected to perform a lymphadenectomy around the CHA. The pancreas was dissected from the superior mesenteric vein (SMV) and transected using a triple-row stapler. Following division of the gastrocolic ligament, the splenic artery was ligated at the splenic hilum, followed by division of the origin of the splenic vein. Subsequently, lymphadenectomy around the SMA was performed toward its origin. The left renal vein was exposed, and retroperitoneal dissection was performed with resection of the anterior Gerota’s fascia. In this case, the left adrenal gland was preserved (level II dissection).^1^ The spleen was dissected with the splenocolic ligament division. Finally, the origin of the CA was dissected and divided (Fig. 2B). At this stage, the specimen was connected only to the CHA and LGA.

For arterial resection and reconstruction, the CHA and LGA were clamped and divided. Frozen section analysis confirmed negative perivascular nerve margins, after which the specimen was resected. Reconstruction of the CHA with the LGA was performed by creating an end-to-end anastomosis using a 6-0 Prolene suture (Fig. 2C). The arterial reconstruction time was 7 min.

After reconstruction of the CHA and LGA, arterial blood supply was confirmed in real time on a monitor using an indocyanine green (ICG) fluorescence system (Fig. 2D). Following ICG injection (2.5 mg/body), arterial blood perfusion to the liver and stomach through the reconstructed artery was immediately visualized, confirming adequate blood flow.

The operative time was 215 min, with an estimated blood loss of 35 mL. Postoperative computed tomography images confirmed good arterial blood flow and patency of the reconstructed arteries (Fig. 1B). The patient was discharged on postoperative day 14, with no complications. The pathological findings revealed R0 resection.

## Discussion

This study presented a novel arterial reconstruction of the LGA supplying the rLHA during DP-CAR. DP-CAR with LGA resection can cause various complications, such as ischemic gastropathy, delayed gastric emptying, gastric perforation, and hepatic ischemia.^2^ Therefore, the feasibility of DP-CAR with LGA reconstruction has been explored in recent reports.^3,4^ In DP-CAR with LGA reconstruction, the middle colic artery (MCA) is typically the first choice for arterial grafting, followed by the jejunal artery as a secondary option.^3,4^ Moreover, the usefulness of intraoperative angiography with ICG fluorescence imaging has been shown to evaluate the reconstruction quality during DP-CAR with LGA reconstruction.^4^

Because the present case had an rLHA from the LGA, the LGA should be reconstructed to avoid hepatic and gastric complications following DP-CAR. Although the MCA could be a candidate, we reconstructed the LGA using the CHA. To the best of our knowledge, this is the first description of a surgical technique for arterial reconstruction of the LGA supplying the rLHA during DP-CAR. This technique would have several advantages and disadvantages. The stump between the CHA and LGA was relatively close in this case, making the use of the CHA a simpler and easier alternative to the MCA. In fact, arterial reconstruction time using the CHA was faster than using the MCA.^4^ Moreover, this technique causes no potential risk of transverse colon ischemia postoperatively. When using the MCA, we carefully need to preserve the marginal and straight arteries of the colon.^3^ In contrast, several critical issues should be addressed for the anastomosis of CHA to LGA during DP-CAR. The anastomotic site between CHA and LGA was closer to the pancreatic stump than that between MCA and LGA. Therefore, the postoperative pancreatic fistula may cause severe complications such as the development of pseudoaneurysm or postpancreatectomy hemorrhage. For the purpose of preventing such complications, we covered the pancreatic stump using the omentum, and separated it from the arterial anastomosis. As proximal CHA is often involved by the tumor, the length of distal CHA to be taken may be limited. Furthermore, the negative frozen section for the soft tissue around CHA and LGA to secure the oncologic clearance is necessary to be confirmed intraoperatively. Therefore, the indication for CHA to LGA needs to be carefully selected for the patients with PDAC involving proximal CHA, even if it is technically feasible. Finally, a precise evaluation for vessel diameter measurements is essential for decision-making. In this case, the calibers of CHA, LGA, and MCA were 5.5 mm, 4.5 mm, and 3.0 mm, respectively. Considering the size discrepancies, the best arterial reconstruction should be chosen. Accordingly, we propose that this technique using CHA-LGA anastomosis may serve as an optional approach in DP-CAR with LGA reconstruction. Moreover, intraoperative angiography with ICG fluorescence imaging facilitated real-time perfusion evaluation of the reconstructed artery and related tissue perfusion during DP-CAR.

In conclusion, this study demonstrated the safety and feasibility of our novel arterial reconstruction of the LGA supplying the rLHA during DP-CAR. Thus, this technique may be a candidate for DP-CAR with LGA reconstruction.

## Supplementary Information

Below is the link to the electronic supplementary material.

Supplementary file1 (MP4 262078 kb)
